# Neuroinflammation in Parkinson’s disease: focus on the relationship between miRNAs and microglia

**DOI:** 10.3389/fncel.2024.1429977

**Published:** 2024-07-26

**Authors:** Ke Xu, Yuan Li, Yan Zhou, Yu Zhang, Yue Shi, Chengguang Zhang, Yan Bai, Shun Wang

**Affiliations:** ^1^The Second Clinical Medical College, Heilongjiang University of Traditional Chinese Medicine, Harbin, China; ^2^Department of Acupuncture and Moxibustion, The First Affiliated Hospital of Harbin Medical University, Harbin, China; ^3^Institute of Acupuncture and Moxibustion, Heilongjiang Academy of Traditional Chinese Medicine, Harbin, China

**Keywords:** neuroinflammation, Parkinson’s disease (PD), microRNA (miRNA), microglia, neurodegenerative diseases

## Abstract

Parkinson’s disease (PD) is a prevalent neurodegenerative disorder that affects the central nervous system (CNS). Neuroinflammation is a crucial factor in the pathological advancement of PD. PD is characterized by the presence of activated microglia and increased levels of proinflammatory factors, which play a crucial role in its pathology. During the immune response of PD, microglia regulation is significantly influenced by microRNA (miRNA). The excessive activation of microglia, persistent neuroinflammation, and abnormal polarization of macrophages in the brain can be attributed to the dysregulation of certain miRNAs. Additionally, there are miRNAs that possess the ability to inhibit neuroinflammation. miRNAs, which are small non-coding epigenetic regulators, have the ability to modulate microglial activity in both normal and abnormal conditions. They also have a significant impact on promoting communication between neurons and microglia.

## 1 Introduction

The prevalence of Parkinson’s disease (PD) is observed in the central nervous system (CNS), making it a prominent neurodegenerative disorder. It is clinically distinguished by the presence of tremors at rest, reduced ability to initiate movement, and difficulties maintaining balance. Additionally, individuals with PD may experience cognitive decline and disruptions in autonomic functions (Tolosa et al., 2021). PD is currently posing a significant challenge to contemporary society (Elsworth, 2020). The key pathological features of PD encompass the degeneration of dopaminergic (DA) neurons and the formation of Lewy bodies (LB) within the substantia nigra (SN) (Munhoz et al., 2024). The main component found in LB is α-Synuclein (α-Syn), and the transformation of α-Syn into oligomers and fibrils acts as a distinguishing characteristic in neurodegenerative conditions like PD (Hamidpour et al., 2024). However, further investigation is imperative to elucidate the cellular and molecular mechanisms underlying the initiation and progression of PD. Specifically, non-coding RNA molecules such as microRNA (miRNA) play a pivotal role in the pathological advancement of α-Syn disease by facilitating the transfer of α-Syn through exosomes (Chandran et al., 2024). For these reasons, exosomes are considered key players in the aggregation and proliferation of proteins in α-Syn disease (Saadh et al., 2024). Exosome-mediated interactions between microglia and DA cells are the key to PD (Meccariello et al., 2023). Exosomes originating from activated microglia may act as vital carriers of α-Syn, resulting in the deterioration of DA neurons and triggering apoptosis in neuronal cells. Hence, microglia have a notable impact on neuroinflammation and initiation of immune system activation beyond the CNS (Chen et al., 2024).

Recent studies have presented findings that support the involvement of activated microglia in promoting the initiation and advancement of PD. However, there is still a lack of clarity regarding the precise relationship between exosomes, microglia, and DA neurons. Exosomes facilitate the transfer of biomolecules (such as RNA and proteins) to remote cells, enabling them to execute their respective biological roles. These exosomes are particularly responsible for transporting miRNAs associated with neurodegeneration, thereby triggering the activation of microglia (Yang et al., 2023). The progression of PD is significantly influenced by the activation of microglia through mechanisms involving exosome-mediated pathways (Guo et al., 2021). Activated microglia, in turn, secrete exosomes that target distant DA neurons, and DA neurons inteRNAlize these exosomes, aggravating the functional changes of DA neurons and ultimately leading to PD progression(Li et al., 2021).

## 2 Microglia in PD neuroinflammation

**FIGURE 1:**
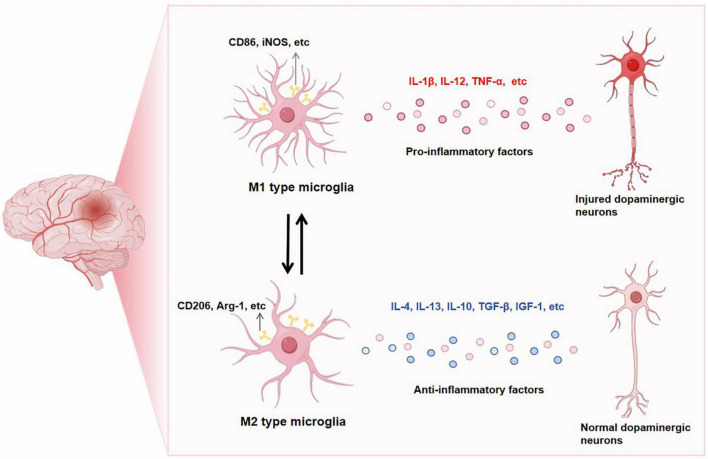
CD86, iNOS, etc. are markers of M1-type microglia. When M1-type microglia is activated, pro-inflammatory factors such as IL-1β, IL-12 and TNF-α are released, leading to the occurrence of PD neuroinflammation. CD206, Arg-1 is a marker of M2 microglia. When M2 microglia are activated, they release anti-inflammatory factors such as IL-4, IL-13, IL-10, TGF-β and IGF-1, which play a neuroprotective role. After intervention, M1 and M2 can be transformed into each other.

Emerging indications propose that the involvement of neuroinflammation is pivotal in the pathological advancement of PD (Araújo et al., 2022). PD is distinguished by the existence of activated microglia and elevated levels of proinflammatory factors, which have a pivotal role in its pathology. Additionally, the activation of microglia acts as a substantial marker for PD (Kam et al., 2020). The cerebrospinal fluid and brain of individuals with PD have shown the presence of proinflammatory mediators, such as TNF-α, IL-1β, and IL-6, particularly in the striatum. This observation provides supporting evidence for the involvement of inflammation in PD (Liu T. W. et al., 2022). The elevated cytokine levels are a consequence of the activation and proliferation of microglia, which occurs early on, is accompanied by neurodegeneration, and persists throughout the course of PD (Isik et al., 2023). When microglia are activated and initiate neuroinflammation, they can display either an M1 phenotype associated with neurotoxicity or an M2 phenotype associated with neuroprotection. Markers of M1 microglia include CD86, iNOS, etc., and markers of M2 microglia include CD206, Arg-1, etc., (Ho, 2019). In the context of activated microglia resembling M1 cells, these cellular entities typically exhibit a shape reminiscent of amoebas, possess an augmented ability to engulf and eliminate dying cell fragments, and release significant amounts of proinflammatory signaling molecules such as IL-1β, IL-12, TNF-α (Colonna and Butovsky, 2017; Borst et al., 2021). The secretion of these factors by microglia is commonly linked to the degeneration of DA neurons in PD (Takahashi and Mashima, 2022). On the contrary, microglia that are activated in an M2-like manner display elongated cell bodies and extensively branched processes. They secrete a range of anti-inflammatory cytokines such as IL-4, IL-13, IL-10, TGF-β, and IGF-1 to attenuate inflammation and facilitate the reparative process (Tang and Le, 2016; Madore et al., 2020). Therefore, the involvement of microglia in inflammation can have both positive and negative effects on disease progression. While the inflammatory response may contribute to neuronal survival, it is important to note that the production of neurotoxic substances could also potentially worsen neurodegeneration (Woodburn et al., 2021; Liu S. et al., 2022; Hasel et al., 2023; Figure 1). Different toxins, including 6-OHDA, MPTP (1-methyl-4-phenyl-1,2,3,6-tetrahydropyridine), and rotenone, as well as transgenic models based on α-Syn, have shown significant microglial activation in various PD models (Lee et al., 2019; Parra et al., 2020; Zhao et al., 2021; Oliynyk et al., 2023). Early occurrence of microgliosis in α-Syn transgenic models precedes the death of DA neurons (Gu et al., 2021). These findings imply that signals triggering the proliferation and inflammation of microglia may be released from toxic aggregates of α-Syn protein or degenerating neurons. The progression of PD is closely linked to the dynamic regulation of microglia (Coomey et al., 2020).

## 3 miRNAs in exosomes

miRNAs, which are small RNAs that are single-stranded and non-coding, have been conserved throughout evolution (Correia de Sousa et al., 2019). The miRNAs play a pivotal role in post-transcriptional gene regulation, exerting a profound impact on cellular growth and development. Moreover, they actively participate in diverse physiological processes including cell proliferation, differentiation, senescence, and apoptosis (Ho et al., 2022; Jie et al., 2022). To date, more than 100 unique miRNA molecules have been detected in immune cells, exerting a vital influence on the regulation of innate and adaptive immune responses’ progression and effectiveness. Moreover, owing to their inherent characteristics as biologically active small compounds, miRNAs possess the remarkable capability to effortlessly traverse the blood-brain barrier (Yang et al., 2021). Hence, miRNAs are one of the material bases for constructing central-peripheral inflammatory networks, creating critical communication pathways between central and peripheral inflammatory states (Diener et al., 2022).

**FIGURE 2:**
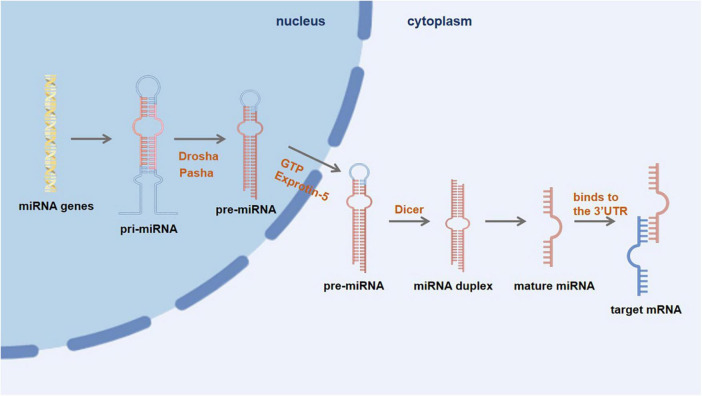
First, miRNA genes are transcribed into primary miRNA (pri-miRNA) within the nucleus. Then, under the action of nuclease Drosha and its cofactor Pasha, pri-miRNA is further processed to form a precursor miRNA (pre-miRNA), which is then transported into the cytoplasm via the GTP-dependent Exprotin-5 complex. Finally, the pre-miRNA is further cleaved by Dicer enzyme to form double-stranded miRNA, and then one miRNA chain is degraded, and another mature miRNA chain binds to the 3′UTR of the target mRNA to degrade or inhibit translation of the target mRNA.

Exosomes primarily consist of miRNAs, a highly representative type of nucleic acids. These exosomes undergo endosome maturation, during which they accumulate additional luminal vesicles. Eventually, the matured endosome fuses with the cell’s plasma membrane and releases its contents, including exosomes, into the extracellular space (Lu and Rothenberg, 2018; Figure 2). While exosomes have the ability to merge with the outer membrane of recipient cells and discharge their contents directly into the cytoplasm, it is more frequently observed that exosomes are taken up by recipient cells through endocytosis (Gerasymchuk et al., 2020). Specifically, microglia has the ability to internalize exosomes through micropinocytosis. Given the phagocytic nature of microglia, it tends to selectively endocytose vesicles present in the extracellular environment. This selective uptake by microglia subsequently influences the transformation of M1 and M2 phenotypes in microglia (Gierlikowski and Gierlikowska, 2022). Microglia is essential in maintaining healthy brain homeostasis and promoting neuropathology (Meldolesi, 2021). Studies have shown that miRNAs act as short, non-coding epigenetic regulators and epigenetic regulation regulates the behavior of microglia under physiological and pathological conditions (Ceccarelli et al., 2021). miRNAs have a crucial regulatory function in microglia during the immune response. The excessive activation of microglia, persistent neuroinflammation, and abnormal polarization of macrophages in the brain can be attributed to the dysregulation of certain miRNAs. Additionally, specific miRNAs have the potential to inhibit neuroinflammation (Oyarce et al., 2022).

## 4 miRNAs associated with pro-inflammatory M1-type microglia in PD

### 4.1 miR-155

In the context of PD, microglial expression of α-Syn and Induced nitric oxide synthase (iNOS) is dependent on the presence of miR-155 (Rastegar-Moghaddam et al., 2023). The induction of suppressor of cytokine signaling 1 (SOCS1), provides feedback to STAT1, is also observed in response to STAT1 (Cianciulli et al., 2017). To examine the impact of DJ-1 on the regulation of SOCS1 suppressor, microglia obtained from the brains of mice with normal DJ-1 expression (WT) and mice lacking DJ-1 (KO) were employed in this investigation. The presence of DJ-1-KO cells resulted in a decrease in the stability of SOCS1 mRNA, which was found to be mediated by miR-155. Interestingly, IFN-γ treatment led to an upregulation of miR-155 expression specifically in DJ-1-KO cells, while no such elevation was observed in WT cells. Reduced levels of DJ-1 led to an upregulation in miR-155 expression, resulting in a downregulation of SOCS1 expression. These results suggest that the promotion of inflammation is facilitated by miR-155, whereas DJ-1 inhibits the expression of miR-155 in microglia treated with IFN-γ to maintain adequate levels of SOCS1 and elicit an anti-inflammatory reaction (Kim et al., 2014).

In a live model of PD induced by adeno-associated virus-mediated α-Syn expression, miR-155 showed a significant rise when examined using an array-based technique. The scientists noted a decrease in the inflammatory reaction to α-Syn and a prevention of α-Syn-induced neurodegeneration when miR-155 was completely eliminated in mice. “In primary microglial mice treated with miR-155, a notable decrease in the inflammatory reaction to α-Syn fibrils was noted, accompanied by reduced levels of major histocompatibility complex II (MHCII) and proinflammatory iNOS expression. Reinstating the inflammatory reaction to α-Syn fibrils was achieved by administering a synthetic miR-155 mimic to these microglia. The findings indicate that miR-155 assumes a pivotal function in the brain’s inflammatory reaction to α-Syn and neurodegeneration associated with α-Syn. These effects may be attributed, to some extent, to the impact of miR-155 on microglia’s reaction towards α-Syn (Thome et al., 2016).

In the BV-2 microglia model, miR-155 was observed to facilitate the upregulation of inflammatory mediators including NO, iNOS, IL-1β, COX-2, and PGE2 in response to lipopolysaccharide (LPS) stimulation. Baicalin effectively reduces neuroinflammation caused by LPS in BV-2 microglia through the inhibition of TLR4-mediated signaling pathways, including TLR4/MyD88/NF-κB and MAPK pathways. Additionally, it attenuates the expression of proinflammatory factors induced by miR-155 (Li et al., 2022).

### 4.2 miR-155-5p

Following TLR activation, there is an observed correlation between NF-κB activity and miR-155-5p. The stimulation of preformed fibrils (PFF) of α-Syn induces the activation of TLR/NF-κB signaling, leading to the transcriptional upregulation of miR-155-5p. The overexpression of miR-155-5p additionally acts as an enhancer, stimulating NF-κB activity by inhibiting SHIP1 expression, thereby inducing a robust release of proinflammatory cytokines. The results suggest that the involvement of miR-155-5p is essential in triggering the inflammatory reaction to PFF within primary microglia. Triptolide interfered with the suppressive impact of miR-155-5p on SHIP1, resulting in a reduction in the phosphorylation levels of downstream components including PI3K, Akt, IKKα/β, and NF-κB. By manipulating the miR-155-5p/SHIP1/NF-κB pathway in primary microglia, triptolide demonstrated inhibitory effects on PFF-induced inflammation. Inhibition of excessive proinflammatory cytokine release can be achieved through attenuation of NF-κB activity and suppression of TNF-α and IL-1β secretion, thereby maintaining a balanced level of NF-κB activity and preventing an overwhelming increase in miR-155-5p levels (Feng et al., 2019).

### 4.3 miR-29b2/c

The activation of glial cells in MPTP-induced PD mice can be enhanced by the expression of miR-29b2/c. After a three-day administration of MPTP, WT mice showed an evident elevation in astrocyte levels along the nigrostriatal pathway, while no concurrent augmentation was detected in the substantia nigra pars compacta (SNpc) of miR-29b2/c KO mice. In addition, the density of microglia in mice lacking miR-29b2/c was noticeably reduced upon the introduction of MPTP. Furthermore, the absence of miR-29b2/c mitigated the detrimental effects of MPTP on both the DA system and glial activation within the nigra striatal pathway. Furthermore, it inhibited the release of inflammatory markers in mixed glial cells exposed to 1-methyl-4-phenylpyridine (MPP), primary astrocytes, or primary microglial cells stimulated with LPS. Therefore, it can be concluded that miR-29b2/c exhibits a proinflammatory role in PD (Bai et al., 2021).

### 4.4 miR-3473b

Research has demonstrated that miR-3473b exhibits a proinflammatory function in PD. In experimental conditions, the suppression of miR-3473b effectively reduced the release of inflammatory factors (TNF-α and IL-1β) triggered by LPS in BV-2 cells obtained from mice. Additionally, it concurrently promoted autophagy in these cells. In an animal study, the administration of miR-3473b antagomir effectively suppressed microglial activation in the SNpc of C57BL/6 mice exposed to MPTP, leading to increased autophagy. Amongst receptors expressed in microglia, TREM2 exhibits exceptionally high levels. Loss of TREM2 is associated with the initiation and progression of PD, while the absence of ULK1 can exacerbate the pathological response in PD. According to Targetscan’s predictions, miR-3473b is predicted to target both TREM2 and ULK1. Furthermore, transfection of miR-3473b mimics has shown its ability to suppress the expression levels of both TREM2 and ULK1. By targeting the expression of TREM2/ULK1, miR-3473b has the potential to modulate the release of proinflammatory signaling molecules (Lv et al., 2021).

### 4.5 miR-132-3p

GLRX is a compact protein that facilitates REDOX reactions of disulfide bonds in interconnected systems, relying on the presence of glutathione. In the PD model, neurodegeneration is worsened by a lack of GLRX (Matsui et al., 2020). In individuals with PD and in cellular models, the expression of miR-132-3p was found to be elevated while the expression of GLRX was observed to be reduced. The interaction between miR-132-3p and GLRX was confirmed using RNA immunoprecipitation and a dual luciferase reporter assay. The levels of TNF-α, IL-1β, and IL-6 in the supernatant of BV-2 cells were found to be higher in the miR-132-3p mimic group compared to the mimic NC group. Furthermore, the potential induction of microglial activation and an inflammatory response may be attributed to the increased expression of miR-132-3p. The effects of miR-132-3p and GLRX on cell survival, programmed cell death, and inflammation were confirmed in BV-2 cells. Furthermore, researcher observed Iba-1 activation and tyrosine hydroxylase depletion in the SNpc region of a mouse model with PD (Gong et al., 2022).

### 4.6 miR-137

The potential of the lncRNA OIP5-AS1 to mitigate the accumulation and detrimental impact of α-Syn induced by MPP in human neuroblastoma SH-SY5Y cells suggests its possible role in the advancement of PD. A cellular model resembling PD (MPP group) was established. Researcher generated an overexpression vector, oe-OIP5-AS1, which was subsequently introduced into MPP ± induced SH-SY5Y cells along with either a miR-137 mimic or si-NIX plasmid. The expression of OIP5-AS1 was found to be downregulated in MPP cells. Upon the overexpression of OIP5-AS1, there was a decrease in miR-137 expression and an increase in NIX expression. Furthermore, the MPP cells showed a reduction in the levels of inflammatory factors and chemokines. It is worth noting that there is an established association between OIP5-AS1 and miR-137, while miR-137 demonstrates a targeting relationship with NIX. Following the overexpression of OIP5-AS1, increased expression of miR-137 or down-regulation of NIX hindered mitophagy and reduced levels of ROS, thereby exacerbating mitochondrial vacuolization. The upregulation of OIP5-AS1 partially counteracted the enhancement of mitophagy and the protective impact on MPP + cells. The promotion of NIX expression and the protection of neurons against degeneration are facilitated by long non-coding RNA OIP5-AS1, which competes with miR-137 for binding, leading to the induction of mitophagy (Zhao et al., 2022) (Table 1).

**TABLE 1:** Summary table of miRNAs associated with pro-inflammatory M1-type microglia in PD.

miRNAs	Related pathways	Related molecular changes	References
miR-155	DJ-1/miR-155/SOCS1	SOCS1↓, STAT1↑	Kim et al., 2014
	miR-155/α-Syn	α-Syn↑, MHCII↑, iNOS↑	Thome et al., 2016
	TLR4/MyD88/NF-κB	NO↑, iNOS↑, IL-1β↑, COX-2↑, PGE2↑	Li et al., 2022
miR155-5p	miR155-5p/SHIP1/NF-κB	PI3K↑, Akt↑, IKKα/β↑, NF-κB↑	Feng et al., 2019
miR-29b2/c	AMPK/NF-kB	IL-1β↑, IL-6↑, TNF-α↑, COX-2↑, IGF-1↓	Bai et al., 2021
miR-3473b	miR-3473b/TREM2/ULK1	TREM2↓, ULK1↓	Lv et al., 2021
miR-132-3p	miR-132-3p/GLRX	GLRX↓, IL-1β↑, IL-6↑, TNF-α↑	Gong et al., 2022
miR-137	miR-137/NIX	NIX↓, Δψm↓, ROS↑	Zhao et al., 2022

## 5 miRNAs associated with anti-inflammatory M2-type microglia in PD

### 5.1 miR-124

The primary mechanism through which extracellular vesicles (EVs) derived from mesenchymal stem cells improve neuroinflammation and neurological function involves the direct modulation of microglial activation in conditions associated with PD (d’Angelo et al., 2020). The existence of miR-124 within EVs has the capability to alter the polarization of microglia from a state that promotes inflammation to one that promotes anti-inflammatory responses. Upon binding to its ligand (LPS), miR-124 triggers microglial polarization and initiates signaling cascades in cells through the Toll-like receptor 4 (TLR4) pathway, which can be either MyD88-dependent or MyD88-independent. The MyD88-dependent pathway recruits TRAF6 factors and IRAKs kinase, leading to the subsequent stimulation of TAK1 kinase. TAK1 promotes the phosphorylation of the IKKβ complex, resulting in the activation and migration of NF-κB transcription factor to the nucleus. This process triggers the induction of proinflammatory genes, including IL-1β, IL-6, IL-18, and TNF-α. Administration of EVs enriched with miR-124 led to a reduction in the expression levels of TLR4, MyD88, IRAK1, TRAF6, and NF-κB p65 in both rats with PD and microglial cell cultures that were stimulated with LPS. Suppression of the TLR4/MyD88 pathway was observed (Izquierdo-Altarejos et al., 2024). The MPTP-induced model of PD exhibited elevated levels of MEKK3 and p-p65 expression, along with microglial activation in the midbrain. The apoptosis and cell mortality in SH-SY5Y cells were effectively suppressed by the upregulation of miR-124 in BV-2 cells upon exposure to the transfer model of microglial culture supernatant. Suppression of MEKK3 and p-p65 expression was observed upon delivery of exogenous miR-124, resulting in decreased microglial activation in mice with MPTP-induced damage to the SNpc. Additionally, miR-124 exhibited a protective effect against apoptotic cell death in midbrain DA-producing cells caused by MPTP in a PD model. Furthermore, it effectively mitigated the upregulation of proinflammatory cytokines induced by LPS and enhanced the secretion of neuroprotective factors. By targeting the MEKK3/NF-κB signaling pathway, miR-124 effectively reduces the inflammatory response induced by microglial activation (Yao et al., 2018). In the mouse model of PD induced by MPTP, there was a notable elevation in the levels of p62 and p-p38 in BV-2 cells, a microglial cell line, following treatment with LPS. Suppression of p62 expression resulted in reduced release of proinflammatory cytokines and p-p38 activation in microglia. Inhibition of p38 activity led to a decrease in the release of proinflammatory cytokines and enhanced autophagy in BV-2 cells. The investigation unveiled a unique role of miR-124 in the modulation of microglial inflammatory reactions through direct interaction with p62 and p38 in PD. In an experimental model where microglia culture supernatant was transferred, the introduction of exogenous miR-124 effectively suppressed the expression of both p62 and p-p38 in the SN region of mice treated with MPTP. This resulted in a decrease in microglial activation. Studies have suggested that miR-124 can inhibit neuroinflammation during PD development by targeting p62, p38, and autophagy (Yao et al., 2019). After exposure to the supernatant obtained from BV-2 cells treated with LPS, SH-SY5Y cells exhibited a notable decrease in their viability and an elevation in apoptosis rate. This was accompanied by an upregulation of circHIPK three expression and a downregulation of miR-124 levels. The upregulation of circHIPK 3 resulted in an elevation in the release of IL-6, IL-1β, and TNF-α from BV-2 cells. In addition, there was an observed increase in the levels of protein expression for markers of microglia (CD11b and Iba-1), as well as an upregulation of factors associated with pyroptosis, namely NLRP3, caspase-1, and ASC following the expression of circHIPK three. The effects were completely reversed upon the introduction of miR-124. Moreover, the results obtained from Western blot and qRT-PCR analyses demonstrated that overexpression of miR-124 effectively suppressed both STAT 3 protein and mRNA expression. Contrarily, cells with silenced miR-124 exhibited increased expression levels of STAT 3 in both mRNA and protein formats (Zhang et al., 2023).

### 5.2 miR-124-3p

The objective of this study was to investigate the expression levels of miR-124-3p and HOXA11-AS in a mouse model with MPTP-induced PD. By analyzing the lncRNA-miRNA-mRNA regulatory network, the researchers utilized the Starbase database to identify that HOXA11-AS directly interacts with miR-124-3p. Researcher observed that HOXA11-AS facilitated MPTP-induced neuronal damage in SH-SY5Y neurons and triggered microglial activation induced by LPS, whereas miR-124-3p exerted an opposing influence. To confirm the targeted interaction, dual luciferase reporter assays were conducted in both SH-SY5Y neurons and BV-2 cells. The results demonstrated a notable reduction in the luciferase activity of HOXA11-ASWT and FSTL1WT when miR-124-3p was introduced. Based on the results, it can be inferred that miR-124-3p is a crucial target of both HOXA11-AS and FSTL1. HOXA11-AS overexpression resulted in increased levels of inflammatory factors and FSTL1, NF-κB, and NLRP3 inflammasome expression. Conversely, miR-124-3p counteracted the effects induced by HOXA11-AS. The miR-124-3p/FSTL1/NF-κB axis shows potential in mitigating microglial activation, neuronal apoptosis, nerve injury, and neuroinflammation induced by MPTP in mice (Cao et al., 2021).

### 5.3 miR-223-3p

Enhancement of miR-223-3p expression demonstrates a neuroprotective impact and directly regulates the levels of inflammatory factors in the nervous system (Wei et al., 2022). Studies have suggested a decline in the levels of miR-223-3p expression among individuals diagnosed with PD (Citterio et al., 2023). DLX6-AS1, an antisense transcript of the Distal-less homeobox 6 gene, has been linked to persistent neuronal injury and programmed cell death (Jia et al., 2020). Research findings have suggested that DLX6-AS1 exhibits the capability to selectively attach itself to miR-223-3p, resulting in a decrease in the levels of miR-223-3p expression within HK-2 cells (Tan et al., 2020). In addition, the simultaneous inhibition of DLX6-AS1 and miR-223-3p led to a decrease in microglial cell viability and an increase in both apoptosis and inflammation levels observed in PD. Upregulated miR-223-3p expression was found to alleviate the exacerbation of cellular toxicity and pyroptosis induced by overexpression of DLX6-AS1 in HK-2 cells. Partially mitigating the suppressive effect of DLX6-AS1 depletion on the LPS-induced inflammatory response in BV-2 microglia was observed upon reversing the downregulation of miR-223-3p (Liu L. et al., 2022). Through bioinformatics analysis, it was discovered that miR-223-3p shares a binding site with both GAS5 and NLRP3. The inflammatory response of LPS-activated PD microglia was evaluated by establishing a BV-2 microglia model with suppressed GAS5 expression. The results revealed an elevation in the levels of pro-inflammatory cytokines IL-1β, IL-6, TNF-α, and NLRP3 inflammasome subsequent to LPS stimulation. However, the suppression of these responses was observed upon knockdown of GAS5. On the other hand, miR-223-3p mimics were introduced into microglial cells to establish a model exhibiting enhanced levels of miR-223-3p expression. In addition, the upregulation of miR-223-3p was found to suppress the synthesis of IL-1β, IL-6, TNF-α, and NLRP3 inflammasome in microglia. These results indicate that the inhibition of the inflammatory response in microglia can be effectively achieved by upregulating miR-223-3p expression and downregulating GAS5 (Xu et al., 2020).

### 5.4 miR-7

The levels of α-Syn protein are reduced in neurons when miR-7 is expressed, as it specifically targets the 3′-UTR region of its mRNA. This mechanism triggers the activation of NLRP3 inflammasome by inducing microglial endocytosis and subsequent liberation of lysosomal cathepsin B. To assess the potential neuroprotective impact of miR-7 on DA neurons, researcher administered miR-7 mimics via stereotactic injection in wild-type (WT) mice subjected to subacute MPTP exposure. Repression of microglial activation in IBA-1 was observed following the administration of miR-7 mimics. The effective reversal of the reduction in TH neurons in the SNpc of mice treated with MPTP was noted. The utilization of miR-7 mimics demonstrated significant inhibition on the activation of the NLRP3 inflammasome, caspase-1, and IL-1β synthesis in MPTP-treated mice (Zhou et al., 2016). The investigation focused on the induction of upregulation in small nucleolar RNA host gene 1 (Snhg1) and downregulation in miR-7 within BV-2 cells, as a result of LPS stimulation. The presence of anti-miR-7 further amplifies the impact of Snhg1 on BV-2 cells. In addition, our results indicate that Snhg1 functions as a competing endogenous RNA to regulate the expression of NLRP3, resulting in the stimulation of the NLRP3 inflammasome. miR-7 alleviated the impact of Snhg1 on BV-2 cells. The transfer of microglial cell culture supernatant resulted in a reduction in apoptosis of primary neurons and an increase in caspase-3 activity, as a result of downregulation of Snhg1 and NLRP3 in LPS-stimulated BV-2 cells. Downregulation of Snhg1 led to an upregulation of miR-7, resulting in reduced activation of microglia and NLRP3 inflammasome in a mouse model of MPTP-induced PD. Furthermore, a decrease in the degeneration of DA neurons located specifically in the SNpc region of the midbrain was observed. To summarize, the miR-7/NLRP3 pathway plays a vital role in the neuroinflammatory mechanisms linked to PD (Cao et al., 2018).

### 5.5 miR-let-7a

In the mouse model of PD induced by α-Syn, miR-let-7a expression was found to be reduced, while STAT3 exhibited concurrent activation in SNpc. Similar results were obtained when BV-2 microglia, exposed to α-Syn, were cultured under laboratory conditions. Based on the findings obtained from BV-2 microglia, it can be inferred that miR-let-7a exerts a direct influence on STAT3, indicating that reduced expression of miR-let-7a might contribute to the activation of STAT3 in an α-synuclein-induced mouse model of PD. Suppression of BV-2 microglial activation and proinflammatory cytokine production was observed upon upregulation of miR-let-7a in response to α-Syn. Hence, it was concluded that miR-let-7a suppressed inflammation mediated by microglia through the regulation of STAT3. Finally, the activation of microglia was suppressed in mice by injecting miR-7 mimics into the striatum, leading to a decrease in proinflammatory cytokine production. This resulted in an alleviation of motor deficits and enhancement of spatial memory deficits in PD mice induced by α-syn. These findings provide evidence supporting the regulatory function of miR-let-7a in neuroinflammation induced by microglia (Zhang et al., 2019).

### 5.6 miR-132 and miR-145-5p

NR4A2, a nuclear receptor and transcription factor, displays unique physiological traits. It is widely distributed within the nucleus of the CNS and plays a vital role in regulating DA differentiation, survival, and maintenance (Dong et al., 2016). Significantly, it plays a regulatory role in various genes that are crucial for DA signaling, and its levels of expression are decreased in the brains of individuals with PD as well as older individuals. Additionally, NR4A2 expression has been observed to inhibit the production of proinflammatory molecules in microglia and astrocytes. Consequently, it serves as a protective factor against inflammation-induced death of DA neurons (Decressac et al., 2013). In mice, the absence of the NR4A2 gene leads to a decrease in DA neurons found in both the SN and ventral tegmental area. The neurotransmission within the nigrostriatal pathway in NR4A2-deficient mice heavily relies on various phenotypes of DA neurons, such as TH and AADC. miR-145-5p has been identified as a potential regulator of NR4A2, with its recognition elements located in the 3′-UTR of NR4A2 being responsible for the repression mediated by miR-145-5p. NR4A2 overexpression can reduce neuronal inflammatory and cytotoxic responses by inhibiting the expression of TNF-α in microglia (Jakaria et al., 2019).

### 5.7 miR-150

In patients diagnosed with PD, a significant inverse relationship was found between the levels of miR-150 in the bloodstream and the presence of proinflammatory cytokines. Moreover, the upregulation of miR-150 expression in BV-2 cells demonstrated a significant inhibitory effect on neuroinflammation upon microglial activation by LPS. In BV-2 cells subjected to LPS treatment, the upregulation of miR-150 resulted in a reduction in the secretion of IL-1β, IL-6, and TNF-α. The direct targeting of AKT3 by miR-150 in BV-2 cells is supported by the identification of a complementary sequence to miR-150 in the 3′-UTR region of AKT3. Repression of relative luciferase activity was observed in cells transfected with wild-type AKT3 and overexpressed miR-150, as indicated by additional luciferase assays (Li et al., 2020).

### 5.8 miR-153-3p

To assess the influence of miR-153-3p on neuronal apoptosis, an experiment was performed in SH-SY5Y cells utilizing a mimic of miR-153-3p and a negative control. The results suggested that the upregulation of miR-153-3p alleviated the reduction in cell viability caused by 6-OHDA and reduced the secretion of lactate dehydrogenase (LDH). In addition, the enhancement of miR-153-3p expression impeded cellular programmed cell death. Based on the data from WB, it was observed that miR-153-3p treatment led to a decrease in the quantities of C-Caspase3, Bax, and SNCA. Simultaneously, there was an elevation in the levels of Bcl2 compared to the control group without any intervention. Following this, a co-culture was performed for 24 hours using SH-SY5Y conditioned medium (CM) transfected with miR-NC and BV-2 cells overexpressing miR-153-3p mimic. The expression of miR-153-3p was found to be significantly reduced in the CM 6-OHDA group compared to the Blank group, as indicated by the qRT-PCR results. Reintroducing miR-153-mimicking molecules into BV-2 cells resulted in elevated expression levels of miR-153-3p compared to the CM 6-OHDA + miR-NC group. In the meantime, it was observed that miR-153-3p intervention in BV-2 cells resulted in a reduction of TNF-α, IL-1β, and IL-6 expression levels as validated through qRT-PCR analysis. In addition, it led to a decrease in the levels of MDA and an increase in SOD activity compared to the CM 6-OHDA + miR-NC group. The participation of NF-κB/COX2/iNOS and Nrf2/HO-1 pathways was identified in mediating the progression of PD. According to the analysis conducted by WB, it was noted that miR-153-3p exhibited inhibitory properties on NF-κB phosphorylation in comparison to the CM 6-OHDA + miR-NC group. In addition, a decrease in the levels of COX2 and iNOS was observed, accompanied by an increase in the contents of Nrf2 and HO-1. Therefore, the increased expression of miR-153-3p inhibited neuronal cell apoptosis and decreased inflammation in microglia by blocking the NF-κB/iNOS/COX2 pathway while enhancing activation of the Nrf2/HO-1 signaling cascade. The increased expression of miR-153-3p leads to a decrease in the levels of synuclein alpha (SNCA) gene, thereby impeding its capacity to suppress cell proliferation and induce pro-apoptotic consequences. These results indicate that the targeting of SNCA by miR-153-3p has an impact on neuronal apoptosis and microglial inflammation (Zhang et al., 2022).

### 5.9 miR-17-5p

Neuronal cell viability was altered and paraquat-induced neuronal apoptosis was reversed due to the release of exosomes by microglia. The RNA sequencing data revealed that these exosomes derived from activated microglia contained significant quantities of circZNRF 1. In addition, the mechanism of circZNRF 1 in regulating PD was investigated using bioinformatics methods, and its target was predicted to be miR-17-5p. Furthermore, enhancing the Bcl-2/Bax ratio can exert a protective effect against apoptosis. Bcl-2 was hypothesized to be a downstream recipient of miR-17-5p. The findings indicated that circZNRF 1 exerted an anti-apoptotic function by sequestering miR-17-5p and modulating the interaction between Bcl-2 and DA neurons’ internalized exosomes. These results validate the presence of a novel method of communication between microglia and neurons, wherein circZNRF1 controls the biological progression of PD via miR-17-5p and has a vital function in safeguarding neurons against PQ-induced apoptosis. These findings have the potential to offer fresh strategies for preventing and treating PD (Liu et al., 2023).

### 5.10 miR-181

Recombinant mimics of miR-181a/b/c/d were administered to BV-2 microglia, which were subsequently exposed to LPS. The activation of microglia was mitigated and the expression levels of iNOS, NO, and ROS in PD were downregulated by all members of miR-181. On the contrary, transfection of inhibitors specifically targeting miR-181a/b/c/d was observed to significantly amplify the activation effect of LPS on microglia. The results obtained from the conducted experiments suggested that miR-181 variants played a role in regulating the activation of microglia. The presence of miR-181a/b/c/d was found to result in a reduction in the expression of PKC-δ induced by LPS. In the concurrent administration, the expression of PKC-δ was further enhanced by inhibitors targeting miR-181a/b/c/d. This elevation in PKC-δ activation plays a crucial role in the transmission of immune signals that result in microglial neurotoxicity. These discoveries suggest that miR-181a/b/c/d might aid in inhibiting microglial activation by reducing the expression of PKC-δ (Ye et al., 2018).

### 5.11 miR-190

The effects of miR-190 on inflammation, neuronal damage protection, and potential therapeutic targets were investigated in mouse models with PD induced by MPTP and BV-2 cells. The results suggested that the expression of miR-190 was decreased in LPS-stimulated BV-2 cells. However, when miR-190 was overexpressed, it led to a suppression of proinflammatory factors including iNOS, IL-6, TNF-α, and TGF-β1, while promoting the expression of anti-inflammatory factor IL-10. The luciferase reporter assay confirmed that miR-190 potentially targets NLRP3. In BV-2 cells stimulated with LPS, the expression of NLRP3 was increased. However, when NLRP3 was knocked down, the inflammatory response induced by LPS in BV-2 cells was suppressed. In addition, when the expression of miR-190 was enhanced or the expression of NLRP3 was decreased, a reduction in LPS-induced apoptosis was observed in BV-2 cells. However, the overexpression of NLRP3 negated the inhibitory impact of miR-190 on apoptosis. Ultimately, the increase in miR-190 levels effectively suppressed microglial activation and inflammation in MPTP-induced PD mice. Furthermore, it resulted in a decrease in tyrosine hydroxylase loss within the SNpc region and prevented neuronal damage (Sun et al., 2019).

### 5.12 miR-195

In this research, an *in vitro* model of microglial activation was created using LPS. The expression levels of miR-195 exhibited a decrease in BV-2 cells upon stimulation with LPS, suggesting the potential involvement of miR-195 in the modulation of microglial activation. In addition, miR-195 mimics or inhibitors were used to conduct successful experiments on microglia cells in order to examine the effects of gain-of-function and loss-of-function. The results indicated that the upregulation of miR-195 inhibited the production of proinflammatory cytokines, including iNOS, IL-6, and TNF-α, while enhancing the release of anti-inflammatory cytokines such as IL-4 and IL-10 in LPS-treated BV-2 cells. Increased levels of Rho-associated coiled coil forming protein kinase 1 (ROCK1) were detected in BV-2 cells upon exposure to LPS. Moreover, the downregulation of ROCK1 through the utilization of small interfering RNA resulted in a comparable impact on the regulation of microglial state as observed with miR-195 overexpression. These results indicate that the interplay between miR-195 and ROCK1 is crucial in triggering microglial activation. Based on these findings, directing attention towards miR-195 could offer a potential therapeutic strategy for addressing PD (Ren et al., 2019).

### 5.13 miR-214-3p

Both animal and cellular models of PD exhibit decreased levels of miR-214-3p. Suppression of miR-214-3p exacerbates deficits in motor coordination and activity, along with the decline in DA neurons. Additionally, it promotes increased reactivity in astrocytes and triggers neuroinflammation. After suppressing the expression of miR-214-3p, mice displayed reduced latency in falling from the rotating rod, decreased traversal of grids, slower movement speed in the open field, a noticeable decline in the count of TH-positive neurons, an increased presence of IBA-1 positive microglia cells, and elevated levels of IL-1b, IL-6, and TNF-a. The group with silenced miR-214-3p demonstrated a considerable reduction in the levels of TGF-b1 and IL-10, accompanied by a significant elevation in the protein levels of iNOS and COX-2. Additionally, there was a significant reduction observed in the protein level of BDNF. The potential binding sites of miR-214-3p and NFATc2 were predicted using the Starbase database, and a dual luciferase assay was conducted to confirm the binding relationship between miR-214-3p and NFATc2 (Liao et al., 2023). Previous studies have documented the ability of NFATc2 to efficiently stimulate microglia in neurodegenerative disorders, and its expression is increased in individuals with PD (Quan et al., 2024). The transcriptional activity of NFATc2 is enhanced by PD, leading to the regulation of cytokines and chemokines that contribute to the formation of a neurotoxic inflammatory environment. After the administration of MPTP, the mRNA expression of NFATc2 in brain tissues showed an elevation, which was later repressed by miR-214-3p to hinder the transcription process of NFATc2.

### 5.14 miR-218-5p

The level of miR-218-5p was found to be reduced in the SN of mice exposed to MPTP and BV-2 cells treated with MPP. The MPTP-induced SN of mice exhibited a notable increase in both the number and volume of IBA-1 microglia and CD68 spots. However, the impact of these outcomes was reduced in mice with an increased expression of miR-218-5p. MiR-218-5p overexpression effectively alleviated microglial inflammation, prevented the degeneration of DA neurons, and improved motor function in MPTP-induced models. The RNA sequence and gene set enrichment analysis revealed the downregulation of the type I interferon (IFN-I) pathway in MPTP-induced mice, which was then counteracted by the overexpression of miR-218-5p. Ddx41 was identified as the gene targeted by miR-218-5p, which was validated through luciferase reporter assay. Overexpression of miR-218-5p or inhibition of Ddx41 was found to suppress the IFN-I response and reduce the expression of inflammatory cytokines in BV-2 cells stimulated with MPP-CM. By regulating the Ddx41/IFN-I signaling pathway, miR-218-5p effectively mitigates neuroinflammation caused by microglia, thereby preserving the integrity of DA neurons (Wang et al., 2024). Therefore, the potential therapeutic approach for PD could involve targeting miR-218-5p/Ddx41.

### 5.15 miR-23b-3p

StarBase made a prediction regarding the interaction site between miR-23b-3p and α-Syn. Upon comprehensive analysis of the experimental findings, it was observed that miR-23b-3p specifically targeted α-Syn and exerted a suppressive effect on its expression. Belonging to the lncRNA family is MALAT1. The expression of the MALAT1 gene is increased in neuronal disorders. Moreover, the validation of the interaction between MALAT1 and miR-23b-3p was conducted through a dual luciferase reporter assay. PD models demonstrate an increase in the expression of MALAT1, which contributes to the activation of inflammatory vesicles in microglia. α-Syn interacts with and influences the process of its internalization and transfer between cells, leading to compromised autophagy and inflammation in microglia. Experiments have demonstrated that upregulating the expression of miR-23b-3p exerts inhibitory effects on this inflammatory reaction. Moreover, scientific investigations have yielded findings that suggest the potential of miR-23b-3p in mitigating neuronal apoptosis triggered by neuroinflammation, thereby indicating its substantial role in the development of PD (Geng et al., 2023).

### 5.16 miR-29c-3p

Prior research has indicated that miR-29c-3p displays anti-inflammatory characteristics in models of PD involving animals and neurons (Li J. et al., 2023; Talebi Taheri et al., 2024). However, the complete understanding of the precise roles and regulatory pathways of miR-29c-3p in microglia remains limited. In order to explore PD, researcher employed LPS-stimulated BV-2 cells as a model system to mimic activated microglia. Our findings revealed a decrease in the expression levels of miR-29c-3p in BV-2 cells induced by LPS. The upregulation of miR-29c-3p effectively suppressed the elevation of Iba-1 caused by LPS, along with the release of proinflammatory cytokines. Furthermore, it inhibited both NF-kB activation and TXNIP/NLRP3 inflammasome activation. Repression of miR-29c-3p resulted in a comparable impact to LPS on the inflammatory response of microglia. The presence of NFATc protein is linked to the promotion of axon growth and neuronal reactions, while suppressing NFATc activity hinders the progression of PD. Researcher observed a negative correlation between NFAT5 and miR-29c-3p, and inhibiting miR-29c-3p prevented the aggravation of microglial inflammation by blocking NFAT5. Microglial inflammatory response is impaired through the targeting of NFAT5 by miR-29c-3p, thereby regulating the NLRP3 inflammasome (Wang et al., 2020).

### 5.17 miR-30e-5p

In the Nurr1 cKO mouse model, there was an observed increase in microglia. Furthermore, a correlation was identified between miR-30e-5p and NLRP3, whereby the overexpression of miR-30e-5p led to alterations in the mRNA expression levels of NLRP3. This suggests that the interaction between miR-30e-5p and specific segments within the 3′-UTR of NLRP3 may enhance the degradation kinetics of NLRP3 mRNA. The results suggest that the regulation of NLRP3 expression and microglia-mediated inflammation is influenced by miR-30e-5p, a specific miRNA controlled by Nurr1. Overexpression of miR-30e-5p was observed to partially mitigate the elevation in NLRP3 level induced by Nurr1 deficiency. These findings imply that miR-30e-5p may play a role in modulating the transcriptional regulation of NLRP3 expression, potentially through its interaction with Nurr1. In addition, the statistical analysis carried out in this study revealed a significant correlation between NURR1, miR-30e-5p, and NLRP3 levels observed in monocytes present in the peripheral blood of individuals diagnosed with PD. This discovery provides supporting evidence for the existence of an axis involving Nurr1/miR-30e-5p/NLRP3 within immune cells located at the periphery (Li T. et al., 2023).

### 5.18 miR-330

In this research, a chronic inflammation model was established in microglial cells and an animal model of PD was induced using LPS. Researcher designed and utilized a plasmid or rAAV 2/5-GFP vector to deliver a miR-330 sponge with six binding sites for miRNA. The levels of miR-330 exhibited a gradual and sustained rise within the initial four days following LPS administration, eventually reaching a steady state. Over time, there was an observed progressive enhancement in the sponge-mediated suppression of M1 polarization. The administration of miR-330 sponge led to an elevation in the levels of SHIP 1 and Arg 1, while reducing the movement of NF-κB and the production of iNOS. These findings indicate a possible inhibition of inflammation. The impact of miR-330 and SHIP 1 interaction on LPS-induced chronic inflammation was investigated by employing Rosiptor, an agonist, and 3AC, an inhibitor that specifically targets SHIP 1. An increase in SHIP 1 expression was observed when miR-330 was downregulated, and similar to Rosiptor, the application of a miR-330 sponge successfully inhibited M1 polarization in primary microglia. Additionally, the inhibition of M1 polarization by the miR-330 sponge was counteracted by 3AC. These results indicate that the interaction between miR-330 and NF-κB is mediated by the SHIP 1 signaling pathway, highlighting its significant role in this process. The findings suggest that the employment of miR-330 sponges consistently impedes microglial polarization triggered by LPS, both in laboratory settings and within living organisms. This phenomenon could potentially be ascribed to the downregulation of NF-κB activity by specifically targeting SHIP1 in microglia, offering a hopeful strategy for safeguarding neurological health (Feng et al., 2021).

### 5.19 miR-335

The levels of miR-335 expression showed significant decrease in different PD mimic scenarios, including the stimulation of LPS in BV-2 microglia and the upregulation of wild-type LRRK2. This downregulation was also observed in the serum samples obtained from mice treated with MPTP. Additional verification was conducted in individuals diagnosed with idiopathic Parkinson’s disease (iPD) and those harboring LRRK2 mutations (LRRK2-PD), thereby affirming the possible clinical significance. From a mechanistic perspective, the mRNA of LRRK2 is directly targeted by miR-335. In microglia cell lines BV-2 and N9, miR-335 effectively decreased the expression of proinflammatory genes triggered by LPS, resulting in reduced levels of receptor-interacting protein 1 (RIP1) and RIP3. These two proteins play crucial roles in signaling pathways associated with necroptosis and inflammation. In addition, the activation of ERK1/2 by LPS was suppressed by miR-335. Moreover, the overexpression of miR-335 resulted in a significant reduction in the expression of proinflammatory genes induced by LRRK2-WT. Ultimately, miR-335 was found to decrease the expression of proinflammatory genes triggered by α-Syn in SH-SY5Y neuroblastoma cells. In summary, research has revealed novel functions of miR-335 in microglia and neuronal cells that effectively counteract conventional inflammatory stimuli or excessive LRRK2-Wt expression, thereby mitigating chronic neuroinflammation (Oliveira et al., 2021).

### 5.20 miR-466b-3p, miR-466c-3p, miR-669c-5p

Additional sequencing and bioinformatics examination of miRNAs associated with microglia-derived EVs demonstrated a predominant targeting of PRAK by these miRNAs. The activation of microglia through the PRAK/MK5 pathway is induced by oligomeric α-Syn. GLPG0259, a targeted inhibitor of PRAK, has the potential to reduce microglial activation caused by oligomer α-Syn. By acting as a pivotal factor in microglial activation, PRAK poses a challenge to the distinct conformation of α-Syn. Receiving miRNAs targeting PRAK, recipient microglia were stimulated to exhibit anti-inflammatory properties through the administration of EVs resulting from monomeric α-Syn treatment. The findings from this investigation suggest that the involvement of PRAK is essential in initiating the activation process of microglia. Furthermore, the combination of GLPG0259, an inhibitor of PRAK, with EV-derived miRNAs specifically targeting PRAK exhibits promising potential in mitigating neuroinflammation observed in neurodegenerative disorders like PD. The crucial role of PRAK in microglial activation when exposed to aggregated or monomeric α-Syn is a significant finding of this study. The mechanism underlying this phenomenon involves the transmission of EV-targeted miRNA-466b-3p, miRNA-466c-3p, and miRNA-669c-5p to microglia. Receptor cells may experience an impact due to the transfer of miRNAs associated with extracellular vesicles, which subsequently regulate target genes for the purpose of modulating cellular function. The potential effects of EV-associated miR-466b-3p, miR-466c-3p, and miR-669c-5p on neuroprotection or anti-inflammation could be attributed to their capability in targeting PRAK (Li et al., 2024).

### 5.21 miR-485-3p

qRT-PCR was employed to evaluate the expression of MiR-485-3p in the serum of PD patients. To mimic neuroinflammation during PD progression, microglia BV-2 cells were exposed to LPS treatment. The serum levels of miR-485-3p exhibit a notable increase in PD patients compared to individuals with Alzheimer’s disease and those who are healthy, thereby demonstrating its potential as a diagnostic tool for screening PD. The expression of miR-485-3p was observed to be upregulated in BV-2 microglial cells stimulated with LPS, and inhibiting miR-485-3p led to a decrease in the secretion of proinflammatory cytokines (IL-1β, IL-6, and TNF-α). According to Target Scan’s prediction, it has been suggested that there is a potential interaction between miR-485-3p and the 3′-UTR sequence of FBXO45, indicating the possibility of binding between miR-485-3p and FBXO45’s 3′-UTR region. To verify the accuracy of this prediction, a luciferase reporter assay was performed. The results indicated that upregulation of miR-485-3p led to inhibition of luciferase activity in WT-FBXO45, while downregulation of miR-485-3p resulted in enhancement of luciferase activity in WT-FBXO45. After the introduction of MUT-FBXO45 into BV-2 cells, there was no significant change observed in the relative luciferase activity. This implies that FBXO45 is directly targeted by miR-485-3p. Previous studies have suggested that suppressing the expression of miR-485-3p can effectively hinder the inflammatory response in BV-2 cells. This implies that the therapeutic potential of targeting miR-485-3p, a regulator of neuroinflammation, should be considered for the treatment of PD (Lin et al., 2022).

### 5.22 miR-7116-5p

The miR-7116-5p in microglia was discovered to have an impact on the inflammation observed in the mouse model of PD induced by MPTP. Microglia were observed to take up MPP through organic cation transporter 3 (OCT3) in order to decrease the levels of miR-7116-5p, a specific miRNA that targets TNF-α. MPP specifically boosts the synthesis of TNF-α in microglia by suppressing miR-7116-5p, leading to subsequent activation of inflammation. Furthermore, the upregulation of miR-7116-5p in mouse microglia impedes the synthesis of TNF-α and activation of glial cells, thereby offering additional defense against the degeneration of DA neurons. To summarize, these findings suggest that MPP suppresses the levels of miR-7116-5p in microglia, leading to an elevation in TNF-α production and inflammatory responses, ultimately resulting in impairment of DA neurons (He et al., 2017) (Table 2).

**TABLE 2:** Summary table of miRNAs associated with anti-inflammatory M2-type microglia in PD.

miRNAs	Related pathways	Related molecular changes	References
miR-124	TLR4/MyD88/NF-κB	TLR4↓, MyD88↓, IRAK1↓, TRAF6↓, NF-κB p65↓	Izquierdo-Altarejos et al., 2024
	miR-124/MEKK3/NF-κB	MEKK3↓, NF-κB↓, p-p65↓, iNOS↓, IL-6 ↓ TNF-α↓, TGF-β1↑, IL-10↑	Yao et al., 2018
	miR-124/MAPK	p62↓, p-p38↓, TNF-α↓, IL-1b↓	Yao et al., 2019
	miR-124/STAT3/NALP3	CD11b↓, Iba-1↓, NLRP3↓, caspase-1↓, ASC↓, STAT3↓	Zhang et al., 2023
miR-124-3p	miR-124-3p/FSTL1/NF-κB	FSTL1↓, NF-κB↓, NLRP3↓, IL-1β↓, IL-18↓, IL-6↓, TNF-α↓	Cao et al., 2021
miR-223-3p	miR-223-3p/NRP1	TNF-α↓, IL-1β↓, IL-6↓, NLRP 3↓, Caspase 3↓	Liu L. et al., 2022
	GAS5/miR-223-3p/NLRP3	IL-1β↓, IL-6↓, TNF-α↓, NLRP3↓	Xu et al., 2020
miR-7	miR-7/NLRP3	NLRP3↓, TH↑, caspase-1↓, IL-1β↓	Zhou et al., 2016
		CD11b↓, TNF-α↓, IL-1β↓	Cao et al., 2018
miR-let-7a	miR-let-7a/STAT3	STAT3↓, iNOS↓, TNF-α↓, IL-6↓, IL-1b↓, IL-12↓	Zhang et al., 2019
miR-132	miR-132/NR4A2	DAT↑, VMAT2↑, TH↑, GDNF↑, α-Syn↓	Jakaria et al., 2019
miR-145-5p	miR-145-5p/NR4A2	TNF-α↓, DAT↑, TH↑, AADC↑	Jakaria et al., 2019
miR-150	miR-150/AKT3	IL-1β↓, TNF-α↓, IL-6↓	Li et al., 2020
miR-153-3p	NF-κB/iNOS/COX2; Nrf2/HO-1	C-Caspase3↓, Bax↓, SNCA↓, TNF-α↓, IL-1β↓, IL-6↓	Zhang et al., 2022
miR-17-5p	circZNRF 1/miR-17-5p/Bcl 2	ROS↓, Bax↓, Bcl-2/Bax↑	Liu et al., 2023
miR-181	miR-181/PKC-δ	iNOS↓, NO↓, ROS↓	Ye et al., 2018
miR-190	miR-190/NLRP3	Nlrp3↓,iNOS↓, IL-6↓,TNF-α↓,TGF-β1↑,IL-10↑	Sun et al., 2019
miR-195	miR-195/ROCK1	iNOS↓, IL-6↓,TNF-α↓,IL-4↑, IL-10↑	Ren et al., 2019
miR-214-3p	miR-214-3p/NFATc2	TH↑,IL-1b↓, IL-6↓, TNF-a↓, TGF-b1↑, IL-10↑,iNOS↓, COX-2↓, BDNF↑	Liao et al., 2023
miR-218-5p	miR-218-5p/Ddx41	IL-1b↓, IL-6↓, TNF-a↓, Irf7↓, Ddx60↓, Nlrc5↓	Quan et al., 2024
miR-23b-3p	miR-23b-3p/α-Syn	α-Syn↓	Geng et al., 2023
miR-29c-3p	miR-29c-3p/NFAT5/NLRP3	IL-1b↓, IL-6↓, TNF-a↓, TXNIP↓, NLRP3↓, ASC↓, caspase-1↓, NFAT5↓	Wang et al., 2020
miR-30e-5p	Nurr1/miR-30e-5p/NLRP3	NLRP3↓	Li T. et al., 2023
miR-330	miR-330/SHIP1/NF-κB	iNOS↓, TNFα↓, IL-6↓, IL-12↓, Arg 1↑, TGFβ↑, IL-10↑, IL-13↑	Feng et al., 2021
miR-335	miR-335/LRRK2	RIP1↓, RIP3↓, TNF-α↓, IL-6↓, ERK1/2↓, NF-κB↓	Oliveira et al., 2021
miR-466b-3p; miR-466c-3p; miR-669c-5p	PRAK/MK5	IL-1β↓, TNF-α↓, NO↓, PGE2↓, MCP-1↓, IL-6↓	Li et al., 2024
miR-485-3p	miR-485-3p/FBXO45	IL-1β↓, IL-6↓, TNF-α↓	Lin et al., 2022
miR-7116-5p	MPP/miR-7116-5p	TNF-α↓	He et al., 2017

## 6 miRNAs are involved in the epigenetic mechanism of microglial activation in PD

Just like the development of mature microglia-specific characteristics, the activation and polarization of microglia in PD necessitate epigenetic modifications linked to M1 and M2 phenotypes (Cheray and Joseph, 2018). The differential expression of diverse miRNAs participating in numerous cellular processes, including cell proliferation, differentiation, programmed cell death, and intercellular signaling, enables discrimination between the quiescent state of microglia and their activated M1 and M2 states (Slota and Booth, 2019). In cellular environments, the primary mechanism by which miRNAs control gene expression is through facilitating the degradation of target mRNAs and initiating translational repression (Kobayashi and Singer, 2022). The coordination of miRNA expression within cells can be influenced by regulatory mechanisms operating at both the transcriptional and post-transcriptional levels, as well as through the influence of molecules originating from both internal and external sources (Rani and Sengar, 2022). Export of various miRNAs occurs, and upon identification, neighboring or distant recipient cells uptake them to modulate gene expression (Kang et al., 2022). Emerging evidence suggests that the process of miRNA exosome stacking is not a random occurrence, but rather relies on four potential mechanisms. These mechanisms encompass sphingomyelinase 2, SUMOylation of heterogeneous nuclear ribonucleoprotein, a pathway dependent on the 3′-end sequence of miRNA, and a pathway associated with the miRNA-induced silencing complex (Zhang et al., 2015). EVs have the ability to transport miRNAs, which can either influence the physiological processes of recipient cells or exacerbate their pathological state, depending on the status of the donor cell (Petralla et al., 2021). In recent years, there has been significant research conducted on the regulation of microglia polarization and inflammation in PD, with a particular focus on their role. Additionally, promising results have been observed regarding their potential as biomarkers for PD and miRNAs.

## 7 Exosomal miRNAs are involved in neuron-microglia crosstalk in PD

Microglia, the immune cells residing in the brain, possess multifaceted roles that extend beyond their immunological functions. Furthermore, microglia actively participate in various aspects of maintaining brain equilibrium. Microglia maintain homeostasis in the brain by constantly engaging with neurons and other cells, enabling them to monitor neuronal activity (Kalluri and LeBleu, 2016). This interaction between neurons and microglia is characterized by a bidirectional and often reciprocal relationship. These reciprocal interactions are observed through various mechanisms: (1) direct physical interactions involving synaptic components; (2) secretion of paracrine signaling molecules that can diffuse and affect neighboring cells; (3) neuronal communication facilitated by microglia through gap junctions and prejunctional protein channels; (4) release of EVs for intercellular communication (Huo et al., 2024).

EVs encompass exosomes, microvesicles, and apoptotic bodies. Certain proinflammatory miRNAs present in exosomes have the potential to trigger the activation of PD microglia and facilitate the oligomerization of α-Syn. Additionally, exosomes expedite polymerization kinetics and enhance the aggregation process of α-Syn (Rezaee et al., 2023). In contrast to free α-Syn monomers, the presence of α-Syn oligomers within exosomes facilitates enhanced cellular absorption and more efficient dissemination (Valencia et al., 2022). Prior studies have revealed that exosomes exhibit the capacity to efficiently transfer α-Syn oligomers between neurons, thereby facilitating the aggregation of α-Syn in unaffected neurons and promoting the transmission of pathological synuclein (Dorostgou et al., 2022). Exosomes create a conducive setting for the aggregation of α-Syn and potentially facilitate the formation of α-Syn oligomers (Zhao and Wang, 2019). α-Syn triggers the activation of microglia, leading to enhanced communication between activated microglia and other glial cells. This interaction results in an increased release of bodily fluids and proinflammatory cytokines, intensifying neuroinflammation and the production of ROS in PD. Consequently, neuronal death is promoted, establishing a detrimental cycle that exacerbates PD progression (Lauritsen and Romero-Ramos, 2023; Miao and Meng, 2024). When microglia surface receptors (such as TLR2 or TLR4) interact with soluble α-Syn, it could potentially result in elevated oxidative stress levels and trigger the activation of inflammatory pathways that involve NF-κB and mitogen-activated protein kinase (Bearoff et al., 2023; Soraci et al., 2023), thus facilitating PD advancement. Conversely, anti-inflammatory miRNAs exert an opposing effect by impeding α-Syn oligomerization, suppressing microglia-induced neuroinflammation, and hindering PD progression (Rademacher, 2023).

**FIGURE 3:**
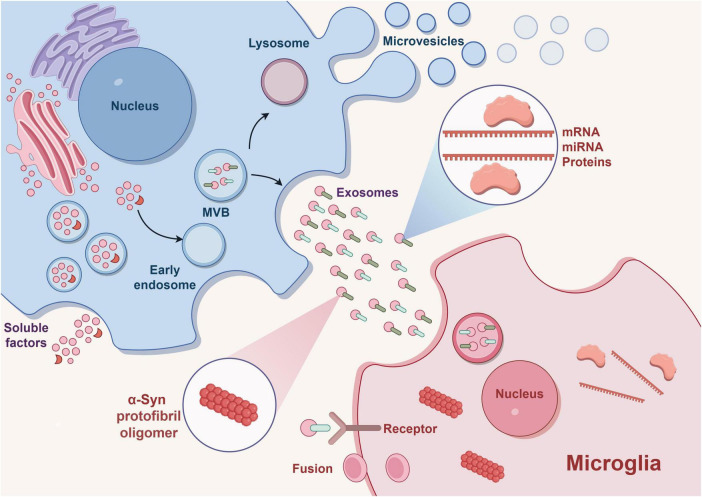
EVs releases exosomes outside the cell and plays a role in transporting proteins, mRNA, miRNA, α-syn, etc. Binding of miRNA to receptors on target microglia leads to phenotypic transformation of microglia and thus affects the occurrence and development of disease. Exosomes can also efficiently transport α-syn oligomers, promoting the transmission of pathologic α-syn. This drawing is done by Figdraw.

Neurons and glial cells, such as microglia, engage in reciprocal communication to ensure proper brain development and functioning. This dynamic interaction is governed by a spectrum of cellular contacts and the secretion of diverse molecules (Deyell et al., 2023; Babu et al., 2024). Both types of cells have the ability to release and receive communication factors through EVs, which facilitate the transportation of proteins, lipids, nucleic acids, particularly miRNAs (Hu et al., 2024). In specific, the phenotype of microglia can be influenced by factors released by neurons, which can either sustain their current state or stimulate their activation. As a result, these factors play various roles in neuroprotection, neuroinflammation, and neurodegeneration (Chen et al., 2022). This alteration in phenotype is induced by a shift in the manifestation of particular microglia genes, which are controlled by epigenetic modifications (including miRNA, DNA methylation, and histone post-translational alterations) generated by EVs (Marangon et al., 2022; Filannino et al., 2024). The microglial phenotype changes with disease progression in all CNS pathological states, especially in chronic neurodegenerative diseases (Davis and Lloyd, 2024). In the initial phase, activated microglia exhibit a neuroprotective function, whereas in the subsequent stage, they contribute to both neuroinflammation and neurodegeneration (Netzahualcoyotzi et al., 2024). While neurodegenerative diseases encompass a wide range of pathologies impacting various brain regions, types of neurons, and aggregates, they all share a common characteristic: the gradual decline in neuronal properties. This decline is accompanied by the activation of microglia and neuroinflammation, which disrupts the interactions between neurons and microglia (Scholz et al., 2024; Figure 3).

## 8 Discussion

Parkinson’s disease is characterized by neuroinflammation caused by microglia, which exhibit both neurotoxic and neuroprotective effects depending on their activation state in the brains of individuals with PD. At present, the majority of research on miRNAs in microglia primarily concentrates on investigating the anti-inflammatory characteristics associated with M2, whereas limited attention has been given to exploring the proinflammatory features linked to M1. The potential impact of miRNAs on the phenotypic transition of microglia extends beyond their ability to decrease the secretion of proinflammatory molecules, and achieving a therapeutic equilibrium between M1 and M2 phenotypes is crucial for evaluation purposes. While reviewing and organizing the literature, it is evident that there are limited studies available on utilizing the M1/M2 ratio as an indicator for therapeutic evaluation. In addition, there have been investigations on the family coherence of certain miRNAs, like miR-181a/b/c/d and their association with microglia. Nevertheless, it is uncommon to find similar studies exploring the family integrity of other miRNAs.

The potential utilization of miRNA delivery systems presents a novel approach to regulate the expression of crucial factors linked to particular diseases. Furthermore, delving deeper into the influence of miRNAs on microglia-associated neuroinflammation may uncover fresh therapeutic targets for intervention. Currently, the therapeutic approaches for miRNA involve the utilization of miRNAs mimics and inhibitors. Additionally, specific immunomodulators have demonstrated their ability to regulate microglial neuroinflammation by disrupting the function of miRNAs. Research is required to discover techniques that can guarantee the precise regulation of downstream miRNAs by miRNAs, in order to prevent any unintended effects. Additionally, when targeting microglia with miRNAs for the treatment of PD, it is crucial to consider and address any adverse effects associated with them. The future research will primarily focus on exploring the delivery mechanism of miRNAs, making efforts to optimize their capacity for impact in the prevention and control of PD.

miRNAs possess significant potential in contributing to the epigenetic process of enhancing disease symptoms through microglial activation. However, the precise interaction between glial cells and neurons is controlled by various mechanisms that still require further investigation using advanced technologies like microfluidic systems, 3D cultures, and *in vivo* imaging. These techniques offer valuable insights into the intricate realm of microglial immune regulation through epigenetic processes and secretion of factors, which necessitate additional exploration.

miRNA-regulated microglia are crucial in the neuroinflammatory process of PD. Neuroinflammation is among the initial pathological alterations observed in PD, and activation of microglia also takes place during the prodromal stage onset of PD. For instance, miR-4639, miR-19b, miR-29a, and miR-29c possess the potential to serve as indicators for detecting the early stage of PD (Wang et al., 2017). Suppose we detect the activation of microglia during the early stage of PD and provide timely intervention. In such a scenario, it is conceivable that we can intervene to impede the onset and progression of PD at its root cause.

## Author contributions

KX: Writing−original draft. YL: Writing−original draft. YaZ: Writing−original draft. YuZ: Writing−original draft. YS: Writing−original draft. CZ: Validation, Writing−original draft. YB: Writing−review and editing. SW: Writing−review and editing.
